# Measuring Menstrual Health Literacy: Development and validation of the Menstrual Health Literacy Scale

**DOI:** 10.1177/13591053261423318

**Published:** 2026-03-23

**Authors:** Sinéad Gallivan, Sinéad Kelleher, Sarah Foley

**Affiliations:** 1 University College Cork, Ireland; 2 Dublin College University, Ireland

**Keywords:** menstrual health, health literacy, psychometric validation, scale development, health psychology

## Abstract

Menstrual health literacy (MHL) is associated with better menstrual health management and greater understanding of how menstruation can impact general health and wellbeing. This article outlines the development and psychometric validation of the 22-item Menstrual Health Literacy Scale (MHLS-22), the first psychometric scale to measure MHL, and proposes a novel conceptual definition of MHL. Development of the MHLS-22 involved initial item generation, content validity analysis, exploratory and confirmatory factor analysis, and convergent and discriminant validity testing. These psychometric analyses provide support for the validity and reliability of the MHLS-22. The MHLS-22 is the first psychometric scale to measure MHL and is suitable for use in the evaluation and development of interventions targeting MHL.

## Introduction

Menstrual symptoms influence both general health status and quality of life (Shimamoto et al., 2021). Research to date has established that pre-existing health conditions, such as migraines, asthma, epilepsy, and rheumatoid arthritis, can be exacerbated during specific stages of the menstrual cycle (Pinkerton et al., 2010). There has also been greater recognition of the impact of the menstrual cycle on mental health, including the prevalence of pre-menstrual dysphoria disorder (PMDD; Marwick et al., 2025). This relationship between menstrual health, overall health status, and health-related quality of life makes it essential for people who menstruate (PWM) to understand and apply accurate information on menstruation when managing their own health.

In recent years, menstrual health literacy (MHL) has been identified as a concept of interest within the wider literature surrounding menstrual health (e.g. Armour et al., 2021). MHL has implications for the management of somatic and psychological menstrual symptoms (Durand et al., 2021; Schoep et al., 2019), help-seeking behaviour and diagnosis of menstrual conditions (Lancastle et al., 2023), and the relationship between the menstrual cycle and quality of life (Hantsoo et al., 2022). Adequate menstrual health knowledge, a key component of MHL, has been previously identified as essential in combatting factors that exacerbate negative experiences of menstruation, such as feelings of shame regarding menstruating, and difficulty in managing menstruation in daily life (Munro et al., 2021). Barrington et al. (2021) further highlight the prevalence of negative wellbeing related to menstruation and identifies stigma, limited access to menstrual resources, and individual burden as antecedents (Barrington et al., 2021). Further, Armour et al. (2021) highlight that low MHL can contribute to the risk of chronic menstrual health conditions going underdiagnosed and undertreated.

Despite the progress in recognising the importance of MHL, the definition of this construct has been inconsistent to date (Hennegan et al., 2020). This study presents a novel conceptual definition of MHL, refining and consolidating previous conceptualisations (e.g. Lopez et al., 2023; McGawley et al., 2023). Building on established multidimensional models of health literacy, this definition acknowledges the personal, social and structural factors that influences an individual’s ability to access and apply health information (e.g. Nutbeam and Muscat, 2021; Pleasant, 2014; Sørensen et al., 2012). It is further informed by definitions in domains of maternal health literacy in pregnancy (Taheri et al., 2020), oral health literacy (Sun et al., 2021), and vaccine literacy (Badua et al., 2022). In defining MHL, it was important to acknowledge the cyclical nature of menstrual health, its relevance across the entire menstrual cycle, and the unique role of communication in MHL compared to other domains of health literacy. For example, although public discourse surrounding menstruation is becoming more commonplace globally, menstrual health remains uncomfortable and taboo for many, with ineffective communication about menstrual health being flagged as a factor contributing to dissatisfaction with medical treatment (Rubinsky et al., 2020).

There is growing acknowledgement of the need for comprehensive interventions to support menstrual health globally. Research to date demonstrates that the on-going work on menstrual health intervention increases knowledge of menstruation (Asumah et al., 2022a). For example, a cross-sectional study of Ghanaian girls reported that two-thirds of school aged girls are knowledgeable about menstrual hygiene practices. However the authors further note that access to menstrual products such as pads remains at 50%, demonstrating the need for further initiatives to support menstrual hygiene (Asumah et al., 2022a) and acknowledgement of both socio-cultural and infrastructural barriers to intervention (Asumah et al., 2022b). Further, a study in the UK reported that almost a quarter of women did not feel comfortable discussing menstrual wellbeing with healthcare providers (GOV.UK, 2022). These constraints placed on menstrual health communication can inhibit a person’s ability to develop their understanding of menstrual experience and knowledge surrounding menstrual health (Armour et al., 2021). The present study, therefore, proposes the following comprehensive conceptual definition of MHL:

Menstrual health literacy refers to the cognitive and social skills informing an individual’s motivation and ability to understand, apply, and communicate accurate information about menstruation, in order to maintain and improve menstrual and general wellbeing throughout the menstrual cycle.

There has been a paucity of research on the measurement of MHL to date (Hennegan et al., 2020). In a systematic review, Hennegan et al. (2020) identified only two psychometric scales (Darabi et al., 2018; Shin et al., 2018) which specifically measured menstrual health. The scales developed in both Shin et al. (2018) and Darabi et al. (2018) were calibrated for adolescent PWM. The Menstrual Health Instrument (MHI; Shin et al., 2018) was designed to be a comprehensive scale of menstrual health, and the Menstrual Health-Seeking Behaviours Questionnaire (MHSBQ-42; Darabi et al., 2018) was developed to assess menstrual health-seeking behaviour. The present study is focussed on the MHL of menstruators of all ages in a global context.

MHL is an emerging area of research and practice. Accordingly, programmes targeting MHL are not yet widespread globally. In populations where such programmes are available, Armour et al. (2021) have observed concerningly low levels of MHL. This finding has given way to calls for the ‘rigorous evaluation’ (Armour et al., 2021: 141) of such interventions. However, as outlined above, no psychometric scale currently exists to measure MHL. Recent years have also seen exploration of the role of digital tools such as apps, in the delivery of accessible MHL interventions (Cunningham et al., 2024). A valid and reliable psychometric scale to measure MHL would further support the development and evaluation of MHL education programmes and interventions, across a variety of settings. Such a scale could be administered at baseline and following a menstrual health intervention, as a statistically robust way of evaluating their efficacy. This approach has proven effective in the area of mental health literacy (Kutcher et al., 2016).

Further, such a scale could potentially be employed in the identification of population-level need for interventions. The use of a MHL scale in developing menstrual health interventions is congruent with the Health Outcome Model (Nutbeam, 2000), which established health literacy as a key outcome of health education and underscored the importance of increased health literacy not only improving knowledge and understanding of health determinants, but also improving attitudes, motivation, and self-efficacy in relation to health behaviours.

The present study aimed to develop a valid and psychometrically robust measure of MHL. The study also presents a novel conceptual definition of MHL, addressing disparities in the definition of core concepts in menstrual health research. The MHLS-22 is the first psychometric scale to measure MHL, addressing calls for more rigorous measurement (Hennegan et al., 2020). In line with recommendations to evaluate existing MHL interventions (Armour et al., 2021), such a measure of MHL may be used to quantify their efficacy and inform the development of future interventions.

## Method and results

The development of the MHL consisted of four phases: item generation, content analysis, exploratory factor analysis (EFA), and confirmatory factor analysis (CFA), followed by testing for convergent and discriminant validity. All statistical analyses were conducted using IBM’s Statistical Software Package for the Social Sciences (SPSS), version 21.0, and AMOS, version 26.0. Ethical approval was received from the School of Applied Psychology Research Ethics Committee, University College Cork (EA-FYP11212023330).

### Phase I: Item generation

Best practices in scale development begin with domain identification (Boateng et al., 2018). This process involves specifying the scale’s purpose and conducting a thorough literature review to ensure that no existing scale serves the same purpose and to identify or inform a conceptual definition (Boateng et al., 2018). This stage of scale development resulted in the definition of MHL outlined above. The initial pool of 65 items for the MHLS was developed through a thematic analysis of a qualitative survey on MHL and a review of relevant literature. Seven participants (*n* = 7), all females aged 25–54 and menstruating, completed the open-ended survey, and the results were analysed using thematic analysis (Braun and Clarke, 2013). Google Scholar, alongside the databases PsycINFO, Cumulative Index of Nursing and Allied Health Literature (CINAHL), Scopus, PubMed, and the National Center for Biotechnology Information (NCBI), were searched for any relevant articles that pertained to MHL.

### Phase II: Content analysis

A panel of six academics in menstrual and women’s health rated the relevance of items included in the initial item pool for clarity and content validity. To assess content validity, the item content validity index (I-CVI) was utilised, a recognised approach that involves expert evaluation in the relevant domain. Experts were invited to review the relevance of each item and provided ratings on a 4-point scale, where one indicated ‘*Not Relevant*’ and four represented ‘*Highly Relevant*’. The ratings of 3 and 4 were aggregated and divided by the total number of raters to compute the I-CVI. Items that achieved an I-CVI greater than a cut-off point of 0.78, as recommended by Polit et al. (2007), were retained for further analysis, while all other items were excluded. This led to the removal of 10 items that fell below the designated threshold, including items such as ‘I want to learn more about the menstrual cycle’ and ‘I believe I know more about periods than my friends do’. Consequently, the 55-item preliminary Menstrual Health Literacy Scale (MHLS-55) was prepared for psychometric analysis.

### Phase III: Scale administration and psychometric analysis

Factor analysis was utilised to develop, refine, and evaluate the structure of the MHLS-55 (Flora and Flake, 2017). In this study, both EFA and CFA were performed on two separate datasets. EFA involves multivariate statistical methods used in scale development to identify latent factors and a posteriori define domains, particularly beneficial for novel scales without pre-existing frameworks (Boateng et al., 2018; Clark and Watson, 2019). As a method of extraction in factor analysis, principal axis factoring (PAF), based on the common factor model, aims to enhance understanding of latent constructs reflected in measured variables. It distinguishes various sources of variances in the data and accounts for random measurement error (Costello and Osborne, 2005; Karami, 2015; Taherdoost et al., 2014). As EFA is used in scale development to identify the latent factor structure within the data (Clark and Watson, 2019), these conceptual distinctions make PAF more suitable for the goals of this research. However, it is important to note that the results of these analyses can often be similar (Karami, 2015). Confirmatory factor analysis (CFA) was employed to test the hypothesised factor structure of the scale that emerged from the exploratory factor analysis (EFA). While EFA is useful for uncovering latent structures without prior assumptions, CFA is essential for evaluating whether the identified factor model provides a good fit to the data in an independent sample. This step is critical in establishing the construct validity of the measure and ensuring that the factor structure is not sample-specific. A well-fitting CFA model provides more substantial evidence for the stability and generalisability of the scale across samples and is a key step in the validation of psychological measures (Brown, 2015).

#### Participants

Two independent samples were recruited in this study, one to conduct EFA and another to conduct CFA and convergent and discriminant validity analyses. Participants for the EFA (*n* = 315) and CFA (*n* = 312) were recruited in March 2024 and December 2024, respectively. Data collection occurred primarily through a combination of physical posters, social networking sites (e.g. Facebook, LinkedIn, Twitter, Instagram), and calls for participants posted on Reddit (e.g. r/periods, r/menstruation, r/psychology, r/inclusivemenstruation) at both time points. The inclusion criteria required participants to be 18 years or older and to identify as someone who menstruates. Gender was not specified in the inclusion criteria, acknowledging that PWM may not necessarily identify as female. Previous studies have identified a lack of data on the menstrual experiences of people who do not identify as cisgender females (Munro et al., 2021). This sample size for both analyses is consistent with recommendations stating that a sample of 300 or more participants is adequate for factor analysis (Clark and Watson, 2019; Comrey and Lee, 2013). The majority of participants for the first point of data collection were residents in Ireland (*n* = 211), the United States (*n* = 43), and the United Kingdom (*n* = 21). An overview of the sample demographics for EFA is provided in Table 1.

**Table 1.** Sample demographics for EFA.

Gender	*n*	%
Male	4	1.3
Female	296	94.0
Non-binary/third gender	13	4.1
Prefer not to say	1	0.3
Self-describe	1	0.3
Age		
18–24	213	67.6
25–34	73	23.2
35–44	22	7.0
45–54	5	1.6

For CFA analysis, data were gathered regarding the diagnosis of menstrual health conditions to enable the assessment of convergent and divergent validity. Again, participants hailed predominantly from Ireland (*n* = 205), the United States (*n* = 28), and the United Kingdom (*n* = 33). A comprehensive overview of the sample demographics for CFA is provided in Table 2.

**Table 2.** Sample demographics for CFA.

Gender	*n*	%
Male	2	0.6
Female	291	93.2
Non-binary/third gender	17	5.4
Prefer not to say	1	0.3
Self-describe	2	0.6
Age		
18–24	127	40.7
25–34	109	34.9
35–44	54	17.3
45–54	22	7.1
Diagnosed menstrual health condition		
Yes	55	17.6
No	201	64.4
I have not been diagnosed but I believe that I have a menstrual health condition	56	17.9

#### Materials

The preliminary MHLS-55 consisted of 55 items, with each item scored on a 7-point Likert scale, ranging from ‘*Strongly Disagree*’ to ‘*Strongly Agree*’. MHLS-55 scores can be calculated by reverse-scoring negatively worded items and then summing item scores. Higher scores indicate greater MHL. This measure was administered at the first point of data collection and used to conduct EFA. The refined MHLS-29 was then administered at the second point of data collection to conduct CFA. The same scoring structure was employed.

#### Exploratory factor analysis

EFA using PAF extraction was then conducted, initially using Kaiser’s criterion (Kaiser, 1960) to produce 16 factors with eigenvalues greater than 1, which accounted for 61.97% of variance. The Kaiser-Meyer-Olkin statistic (KMO = 0.785) indicated sampling adequacy (Taherdoost et al., 2014). Bartlett’s test of sphericity (Bartlett, 1950) was significant (*x*^2^ = 5760.32, df = 1485, *p* < 0.001), indicating that the matrix of correlations was significantly different from an identity matrix and therefore was suitable for EFA (Taherdoost et al., 2014). To simplify the statistical output, small coefficients below 0.4 were suppressed in line with the recommendation of Field (2013) to discard items with loadings below this value. Examination of the scree plot suggested a four-factor solution (see Figure 1).

**Figure 1.**
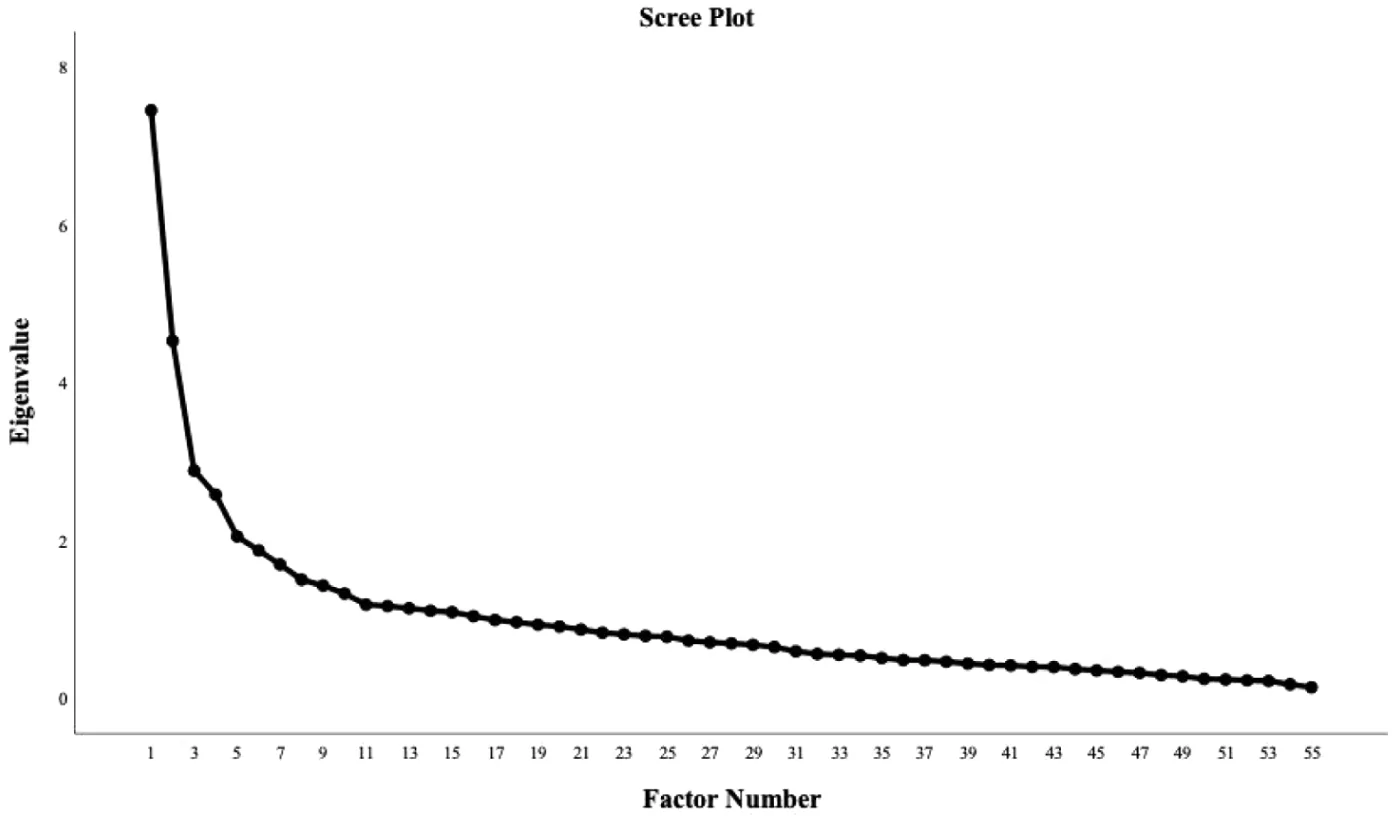
Scree plot.

PAF using a four-factor solution and varimax rotation produced a better factor structure and accounted for 31.71% of the variance. At this stage, the rotated factor matrix was examined for items that did not load onto any factor or exhibited cross-loading. No cross-loading was observed, and 26 items that did not load onto any factor were identified for deletion. Following the removal of these items, PAF with four factors and varimax rotation produced a rotated factor matrix that accounted for 49.54% of the variance. All loadings were greater than 0.4, and no cross-loading was exhibited. Parallel analysis using O’Connor’s (2000) syntax confirmed a four-factor solution. Promax rotation was selected as it produced the solution with the greatest factor loading. The EFA yielded a preliminary 29-item, 4-factor MHLS-29 (Table 3).

**Table 3.** Pattern matrix: principal axis factoring with promax rotation.

Items	Factor 1	Factor 2	Factor 3	Factor 4
	Skills and behaviours	Identifying abnormal symptoms	Beliefs about menstrual health	Communicating about menstrual health
I am able to differentiate between accurate and inaccurate information about menstrual health	0.776			
I know how to access accurate information about my period.	0.692			
If someone asked me questions about the menstrual cycle, I am confident that I could answer them accurately.	0.629			
I am confident that I know how to best manage my period pain.	0.585			
I often get confused when trying to access information about my period	0.573			
I know what questions to ask the doctor about my period.	0.538			
I make sure that I get information about my period from a reliable source	0.526			
I double-check information about the menstrual cycle to make sure that it’s reliable.	0.525			
I know which painkillers are most effective for period pain.	0.495			
I know how much to take when using painkillers to effectively manage period pain.	0.485			
I don’t know whether my period is normal	0.463			
I understand how my period is affected by external factors such as smoking, diet, exercise, stress, weight and pregnancy history.	0.462			
I would speak to a doctor if my periods became a lot heavier or lighter than I’m used to.		0.849		
I would speak to a doctor if my periods became much longer or shorter than I’m used to.		0.847		
I would speak to a doctor if my periods became much more frequent or much more irregular than I’m used to.		0.825		
I would speak to a doctor if I began to experience new period symptoms that I found difficult to manage.			0.64	
I would speak to a doctor if my periods became much more painful than I’m used to.			0.612	
The only important parts of the menstrual cycle are ovulation and menstruation.			0.556	
After you learn how to use pads/tampons and prevent pregnancy, there is little more to learn about menstrual health.			0.547	
Menstrual health is not related to my overall quality of life			0.496	
Beyond pregnancy, there is no reason why I would be concerned if my periods changed all of a sudden (i.e. length, symptoms, frequency)			0.487	
All painkillers are equally effective at managing period pain			0.448	
Menstrual health is an integral part of general health.			0.444	
Your cycle tends to stay the same throughout your whole life until you stop menstruating and reach menopause.			0.64	
I don’t think that period symptoms can impact academic or professional performance			0.612	
I am comfortable talking about menstruation				0.753
I often discuss various aspects of the menstrual cycle with my friends				0.713
I am uncomfortable discussing menstruation publicly				0.699
I am uncomfortable discussing menstruation privately with other people who menstruate				0.635

*Note*. Rotation converged in six iterations.

Factor 1, *Menstrual Health Skills and Behaviours*, consisted of 12 items that focussed on practices and behaviours associated with menstrual health. Factor 2, *Identifying Abnormal Symptoms*, comprised five items pertaining to identifying when to seek medical advice about menstrual symptoms. Factor 3, *Beliefs About Menstrual Health*, included eight items, which related to individuals’ beliefs and attitudes surrounding menstrual health. Finally, factor 4, *Communicating About Menstrual Health*, consisted of four items that addressed PWM’s ability and motivation to communicate with others about the menstrual cycle.

#### Confirmatory factor analysis results

CFA was conducted to confirm the factor structure of the preliminary 29-item, 4-factor MHLI-29 and to test hypotheses derived from EFA, utilising a different dataset. A correlated factors model was applied using SPSS’s AMOS (Arbuckle, 2015), which assessed and validated the EFA structure through maximum likelihood CFA. To achieve model identification, the loadings for the first item of each factor and the item residuals were set to 1.0 (Rhudy et al., 2020).

The initial model (M1) results revealed that several factor loadings were below 0.5 and needed to be removed before assessing model fit (Hamid et al., 2011). Five items were removed from the *Beliefs about Menstruation* subscale and two from the *Skills and Behaviours Subscale*. The model was subsequently rerun excluding these items (Model 2), and the overall fit of the model was assessed through various goodness-of-fit indices. Expected cut-off points for fit indices were as follows: root mean square error of approximation (RMSEA) < 0.05; SRMR < 0.9; AGFI > 0.8, comparative fit index (CFI) > 0.9; CMIN/DF > 3,0; NFI > 0.9; GFI > 0.9 (Boley and McGehee, 2014; Hair et al., 2010; Hu and Bentler, 1999). Several fit indices did not meet the criteria for good model fit.

Therefore, adequate model fit was achieved through model re-specification informed by the modification indices (see Table 4). Thus, the model (M3) was modified by correlating the residuals of the highest modification indices. These modifications significantly improved the resulting CFA model without altering the structure of item factor loadings, and the fit indices met the criteria for good model fit.

**Table 4.** Model indices.

Model	Chi-square (χ^2^)	DF	RMSEA	CFI	CMIN/DF	NFI	GFI	AIC	BIC
Model 1 (M1)	1050.09	371	0.077	0.798	2.83	0.721	0.811	1178.088	1417.84
Model 2 (M2)	728.116	203	0.091	0.814	3.587	0.762	0.820	828.116	1015.43
Model 3 (M3)	317.960	188	0.047	0.954	1.691	0.896	0.916	447.960	691.463

DF: degrees of freedom; M1: initial model; M3: final model.

CFA resulted in a 22-item, four-factor solution: the MHLS-22. Given the large sample size, acceptable measures, and path diagram, it can be concluded that the MHLS-22 has a good model fit (see Figure 2, path diagram).

**Figure 2.**
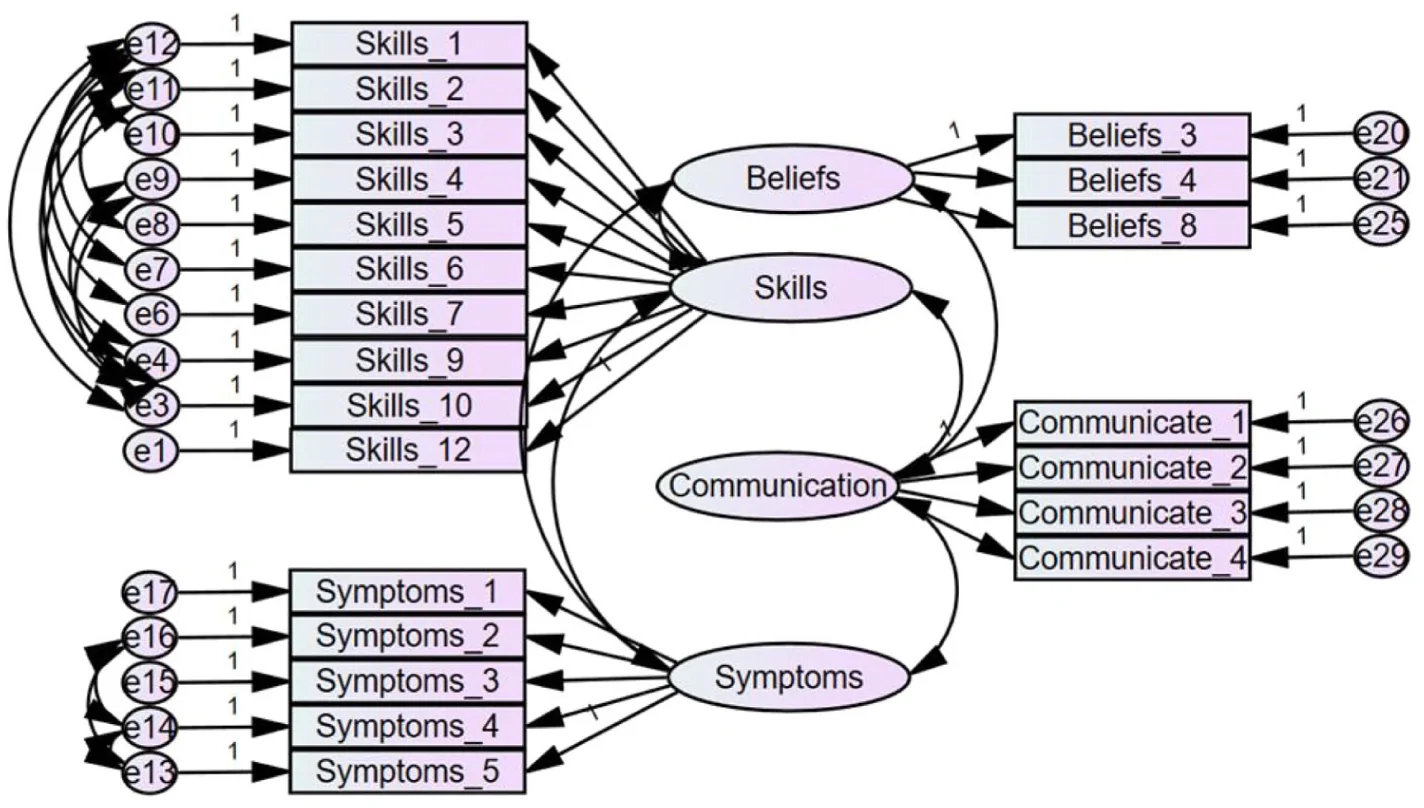
Path diagram.

### Phase IV: Convergent/divergent validity and additional reliability data

It was hypothesised that a significant relationship would exist between the Generalised Self-Efficacy Scale (GSE; Schwarzer and Jerusalem, 1995) and the *Menstrual Health Skills and Behaviours* and *Identifying Abnormal Symptoms* subscales of the MHLS-22. Specifically, that participants who scored higher on these subscales of the MHLI would also possess greater self-efficacy as measured by the GSE. It was also hypothesised that greater scores on the *Menstrual Health Skills and Behaviours* subscale would also be related to greater levels of overall health literacy, as measured by the Health Literacy Survey 2019, 12-item short form questionnaire (HLS_19_-Q12; Pelikan et al., 2022). Moreover, a significant relationship was hypothesised to exist between the MHLI subscales *Beliefs About Menstrual Health* and *Communicating About Menstrual Health* and the Menstrual Attitudes Questionnaire (MAQ; Brooks-Gunn and Ruble, 1980). It was expected that those with more positive menstrual attitudes, as indicated by a higher score on the MAQ, would also have more positive beliefs about menstrual health and more open attitudes towards communicating about menstrual health, as indicated by higher scores on the respective subscales. Finally, it was hypothesised that significant differences exist between groups known to differ in MHL and the diagnosis of a menstrual health condition, with those having a confirmed diagnosis more likely to exhibit better MHL.

#### Measures

*The HLS19-Q12* instrument used in this research was developed within ‘HLS-19 - The International Health Literacy Population Survey 2019-2021 of M-POHL’ (Pelikan et al., 2022): The Health Literacy Scale 2019 short form (HLS19-Q12) is a 12-item version of the HLS19-Q47, a comprehensive health literacy measure validated in the general adult population in 17 European countries (Pelikan et al., 2022). Items are rated on a four-point scale from *‘Very difficult’* to *‘Very easy’*, with an additional option for *‘I don’t know’*. This scale demonstrated very good internal consistency in this sample (α = 0.869).

##### Generalised Self-Efficacy Scale

The Generalised Self-Efficacy Scale (GSE) (Schwarzer and Jerusalem, 1995) is a 10-item scale which measures perceived self-efficacy. Items are rated on a four-point scale from *‘Not at all true’* to *‘Exactly true’*. Excellent internal consistency was demonstrated in this sample (α = 0.878).

##### Menstrual Attitudes Questionnaire

The Menstrual Attitudes Questionnaire (MAQ) (Brooks-Gunn and Ruble, 1980) is a 33-item scale measuring attitudes towards menstruation. The MAQ consists of five subscales: *Menstruation as a debilitating event; Menstruation as a bothersome event; Menstruation as a natural event; Anticipation and prediction of the onset of menstruation*, and *Denial of any effect of menstruation.* Items are rated on a 7-point Likert scale ranging from ‘*Strongly Disagree’* to ‘*Strongly Agree’*. Internal consistency in our sample was modest (α = 0.646), falling just below the conventional threshold of 0.70. (Boateng et al., 2018).

## Results

The correlations presented in Table 5 provide support for the construct validity of the MHLS-22. Correlations between the scales were statistically significant but modest in strength, ranging from *r* = 0.030–0.262 (*p* < 0.05). These results suggest limited overlap between the measures. Despite the modest strength of the correlations, their statistical significance, in combination with strong internal reliability and factor alignment, provides preliminary support for convergent validity. As hypothesised, greater health literacy as measured by the HLS19-Q12 was associated with higher scores on the subscale *Menstrual Health Skills and Behaviours* (*r* = 0.256, *p* < 0.01). Measures of generalised self-efficacy as measured by the GSE, were also associated with higher scores on the subscales *Menstrual Health Skills and Behaviours* (*r* = 0.262, *p* < 0.01) and *Identifying Abnormal Symptoms* (*r* = 0.152, *p* < 0.01). More positive attitudes towards menstruation were also associated with higher scores on the subscales *Beliefs About Menstrual Health* (*r* = 0.201, *p* < 0.01) and *Communicating About Menstrual Health* (*r* = 0.186, *p* < 0.01). To further evaluate construct validity, a known-groups comparison was conducted to examine whether scores on the MHL differed by diagnosis, given theoretical expectations that diagnostic status would be associated with varying skills, knowledge, communication, and other factors. A one-way ANOVA was performed to assess whether MHL scores varied by diagnostic group (no diagnosis, diagnosis, unsure). Diagnosis had a statistically significant effect on scale scores, *F*(2, 309) = 5.35, *p* = 0.005. Post hoc comparisons (Tukey) revealed that participants with a diagnosis had significantly higher scores than those who had not been diagnosed but believed they had a condition (*p* = 0.003), supporting known-groups validity. No significant difference was found between the no diagnosis group and the other groups.

**Table 5.** Correlations.

		Skills	Symptoms	Beliefs	Communication	MAQ	HLS19-Q12	GSE
Skills	Pearson correlation	1	0.351**	−0.196**	0.029	0.078	0.256**	0.262**
	Sig. (two-tailed)		<0.001	<0.001	0.606	0.171	<0.001	<0.001
	*N*	313	313	313	313	313	313	312
Symptoms	Pearson correlation	0.351**	1	−0.256**	0.078	−0.052	0.081	0.152**
	Sig. (two-tailed)	<0.001		<0.001	0.169	0.364	0.151	0.007
	*N*	313	313	313	313	313	313	312
Beliefs	Pearson correlation	−0.196**	−0.256**	1	−0.035	0.201**	0.081	−0.116*
	Sig. (two-tailed)	<0.001	<0.001		0.532	<0.001	0.152	0.040
	*N*	313	313	313	313	313	313	312
Communication	Pearson correlation	0.029	0.078	−0.035	1	0.186**	−0.080	0.030
	Sig. (two-tailed)	0.606	0.169	0.532		<0.001	0.160	0.600
	*N*	313	313	313	313	313	313	312
MAQ	Pearson correlation	0.078	−0.052	0.201**	0.186**	1	−0.066	0.067
	Sig. (two-tailed)	0.171	0.364	<0.001	<0.001		0.246	0.239
	*N*	313	313	313	313	313	313	312
HLQ	Pearson correlation	0.256**	0.081	0.081	−0.080	−0.066	1	0.238**
	Sig. (two-tailed)	<0.001	0.151	0.152	0.160	0.246		<0.001
	*N*	313	313	313	313	313	313	312
GSE	Pearson correlation	0.262**	0.152**	−0.116*	0.030	0.067	0.238**	1
	Sig. (two-tailed)	<0.001	0.007	0.040	0.600	0.239	<0.001	
	*N*	312	312	312	312	312	312	312

** Correlation is significant at the 0.01 level (two-tailed).

* Correlation is significant at the 0.05 level (two-tailed).

The internal consistency of the MHLS-22 subscales was assessed using Cronbach’s alpha. The subscales *Menstrual Health Skills and Behaviours* (α = 0.84), *Identifying Abnormal Symptoms* (α = 0.89), and *Communicating About Menstrual Health* (α = 0.83) all showed excellent internal consistency while the subscale *Beliefs About Menstrual Health* demonstrated acceptable reliability (α = 0.64). All values are within the expected range for psychological constructs, although the lower alpha for *Beliefs About Menstrual Health* warrants cautious interpretation.

## Discussion

### Summary

The present study developed the Menstrual Health Literacy Scale (MHLS-22), a 22-item, four-factor psychometric scale which assesses MHL. The MHLS-22 was developed and refined through multiple stages. First, an initial item pool, informed by a literature review and qualitative data analysis was generated. These initial 65 items were refined following content validity analysis using the I-CVI, resulting in the elimination of 10 items. EFA using PAF was then carried out to identify latent factor structure, resulting in a preliminary 29-item, four-factor scale (MHLS-29). CFA was then carried out on the emergent MHLS-29, resulting in the elimination of seven items for good model fit, yielding the MHLS-22. Factor analysis provided support for the psychometric soundness of the MHLS-22 and its suitability as a theoretically based, multidimensional measure of MHL. This was evidenced through high levels of internal consistency among items, as well as good model fit and acceptable factor loadings.

Correlational analyses provided preliminary support for the convergent and known-groups validity of the scale. The first factor, *Menstrual Health Skills and Behaviours* consists of items relating to an individual’s skills in accessing, understanding, evaluating, and applying menstrual health information. Items relating to pain management capture this element of MHL, in line with the finding in Armour et al. (2021) that many PWM demonstrate insufficient understanding of how to self-manage menstrual pain, indicative of poor MHL. This factor also relates to individuals’ self-efficacy in managing their menstrual cycle and symptoms, for example, ‘I know how to access accurate information about my period’. Self-efficacy is an important component of health literacy and has been identified as a predictor of higher levels of health literacy in other domains (Berens et al., 2022).

The second factor, *Identifying Abnormal Symptoms*, addresses what symptoms would motivate PWM to seek medical advice. Many barriers to seeking menstrual healthcare have been identified in the literature, including negative perceptions of available healthcare (Chen et al., 2018), stigma (Durand et al., 2021), normalisation of abnormal menstrual symptoms (Armour et al., 2021), and previous negative experiences seeking care (Li et al., 2020). MHL can empower PWM to seek necessary care by developing their understanding of abnormal and potentially concerning menstrual symptoms. As PWM can be unsure as to whether a ‘normal’ menstrual cycle or symptom relates to being normal for everyone, or normal for their cycle (Wood et al., 2007), the items in this factor clarify this matter for scale users, for example, ‘I would speak to a doctor if my periods became a lot heavier or lighter than I’m used to’. To address the phenomenon in which PWM feel that their pain has to be extreme in order to warrant help-seeking (Windrim et al., 2024), this factor also includes a more general item, ‘I would speak to a doctor if I began to experience new period symptoms that I found difficult to manage’’.

The items in the third factor, *Beliefs About Menstrual Health*, pertain to misconceptions (e.g. ‘Menstrual health is not related to my overall quality of life’.) about menstrual health. Previous investigations have found that while the individual experience of menstruation is central to shaping attitudes, higher levels of knowledge can mitigate shameful attitudes towards menstruation or negative self-image (Kvalem et al., 2024). This relationship makes beliefs an important aspect to consider when measuring MHL as they may be indicative not only of menstrual knowledge, but of motivation to access, evaluate and apply menstrual health information. The internal consistency of this sub-scale (α = 0.64) implies that items could be further examined in future adaptions of the scale.

The fourth factor, *Communicating About Menstrual Health*, addresses individuals’ attitudes (e.g. ‘I am uncomfortable discussing menstruation publicly’.) and behaviours (e.g. ‘I often discuss various aspects of the menstrual cycle with my friends’.) surrounding talking about menstruation. Communicating about menstrual health is important for PWM in developing an understanding of a normal menstrual experience and menstrual health knowledge (Armour et al., 2021). Cultural taboos have traditionally suppressed open communication surrounding menstruation (Rubinsky et al., 2020), inhibiting the development of MHL.

As the majority of the sample in the present study was Irish, it is important to consider that many Irish PWM report inadequate menstrual health education in school (Ní Chéileachair et al., 2022). This shortfall places an even greater emphasis on the role of communication in MHL as informal discussions with family, friends, and academic mentors about menstrual health may substitute formal education as the primary source of menstrual health knowledge (Armour et al., 2019).

### Limitations

Despite efforts to recruit a varied sample, participants across both samples were primarily young Irish women. Given the homogeneity of our sample, it is important to acknowledge limitations regarding generalisability of the findings. To strengthen the relevance of the MHLS-22, the psychometric properties of the scale need to be examined in more diverse samples (Clark and Watson, 2019). Specifically, future studies should focus on translating and validating the MHLS-22 across culturally and contextually diverse populations, to enhance its’ cross-cultural reliability and conceptual equivalence in non-Western regions (Asumah et al., 2022b). These efforts would ensure the measure remains meaningful across different linguistic and healthcare contexts, including community-based healthcare provision. This is particularly relevant in MHL due to the volume of research focussing on improving menstrual hygiene and management in the global south (e.g. Baird et al., 2022; Holmes et al., 2021). Additionally, attention should be given to potential gendered barriers in healthcare access, particularly the dismissal of women’s symptoms related to menstrual or hormonal health, to improve the measure’s sensitivity to diverse healthcare experiences (Asumah et al., 2022; Shannon et al., 2021). Although the MHLS-22 was designed to capture menstrual health literacy among PWM, we acknowledge that future adaptations should also assess menstrual health literacy in non-menstruating individuals. This may involve examining menstrual health knowledge and attitudes across all genders as well as heterogeneous groups, including healthcare providers. Such adaptations might be achieved through rewording items, as well as making culturally and contextually relevant adjustments, followed by further psychometric testing. Furthermore, while the measure demonstrated robust performance across exploratory and confirmatory factor analyses, future studies should specifically examine differences in psychometric functioning and item properties according to menstrual health conditions (i.e. endometriosis, PCOS) to ensure its validity and reliability across a broad range of menstrual health experiences.

### Implications

The MHLS-22 is the first psychometric scale to measure MHL. MHL in menstruating adults has been overlooked to date, despite previous investigations into the impact of the menstrual cycle on the capacity of this cohort to engage in social activities (Armour et al., 2020; De Sanctis et al., 2016) and professional, academic, and household tasks (Schoep et al., 2019). Prior to this study, psychometric scales used in menstrual health research focussed on female adolescents (e.g. Darabi et al., 2018; Shin et al., 2018). Similarly, much of menstrual health research focuses on the experiences of female adolescents and the stage surrounding menarche (e.g. Evans et al., 2022). The clarity of item wording and use of colloquial language enhances the accessibility of the scale, broadening the scope of its application.

This scale will facilitate clinicians, community-based practitioners and researchers in the evaluation and development of interventions targeting MHL (Armour et al., 2021). The MHLS-22 can be administered before and after MHL interventions to quantify their impact, congruent with the recommendation of Nutbeam et al. (2018) to use valid and reliable measurement tools in systematically evaluating the efficacy of health literacy interventions. This approach has been implemented as standard practice in other domains of health literacy, such as mental health literacy (DeLuca et al., 2021; Wei et al., 2024), resulting in significant advances (Kutcher et al., 2016). The MHLS-22 will therefore support best practice in the development and evaluation of MHL interventions.

This study also proposes a novel definition of MHL. Inadequate and inconsistent definition of key concepts in menstrual health research has been identified as a challenge within the area (Hennegan et al., 2020). This definition of MHL will allow for increased consistency and clarity across studies in the area and will also enrich understanding of the construct. Previously, influential studies in MHL research have referenced general definitions of health literacy in relation to menstrual health (e.g. Armour et al., 2021; Holmes et al., 2021; McGawley et al., 2023). This approach has resulted in some unique aspects of MHL being overlooked in the literature. The present definition of MHL includes communication as an element of MHL due to the specific sociocultural factors that place a greater emphasis on communication in MHL than in other domains of health literacy. As the menstrual cycle can impact how PWM feel and function both during menstruation and across the entire month (Pierson et al., 2021), it was essential that this was reflected in our definition of MHL.

## Supplemental Material

sj-docx-1-hpq-10.1177_13591053261423318 – Supplemental material for Measuring Menstrual Health Literacy: Development and validation of the Menstrual Health Literacy Scale Supplemental material, sj-docx-1-hpq-10.1177_13591053261423318 for Measuring Menstrual Health Literacy: Development and validation of the Menstrual Health Literacy Scale by Sinéad Gallivan, Sinéad Kelleher and Sarah Foley in Journal of Health Psychology

## Data Availability

The data will be made available on the OSF once the original work is published. Consent for participants has been obtained.
