# Supplementary material for: Measuring Menstrual Health Literacy: Development and validation of the Menstrual Health Literacy Scale
Source: J Health Psychol. 2026 Mar 23;31(13):5701–17. doi: 10.1177/13591053261423318 (PMC13683927; doi:10.1177/13591053261423318)
Supplement: Supplementary file 1 [file sj-docx-1-hpq-10.1177_13591053261423318.docx]

## ***Item Content-Validity Indices (I-CVI)***

| Item | **I-CVI** |
| --- | --- |
| *Your cycle tends to stay the same throughout your whole life until you stop menstruating and reach menopause. | 0.83 |
| *The only important parts of the menstrual cycle are ovulation and menstruation. | 0.83 |
| *Beyond pregnancy, there is no reason why I would be concerned if my periods changed all of a sudden (i.e. length, symptoms, frequency) | 0.83 |
| *All painkillers are equally effective at managing period pain | 0.83 |
| *After you learn how to use pads/tampons and prevent pregnancy, there is little more to learn about menstrual health. | 0.83 |
| I am confident that I know how to best manage my period pain. | 0.83 |
| I know how much to take when using painkillers to effectively manage period pain. | 1.00 |
| I would speak to a doctor if my periods became much more frequent or much more irregular than I'm used to. | 1.00 |
| I would speak to a doctor if my periods became a lot heavier or lighter than I'm used to. | 1.00 |
| I would speak to a doctor if my periods became much longer or shorter than I'm used to. | 1.00 |
| I would speak to a doctor if my periods became much more painful than I'm used to. | 1.00 |
| I would speak to a doctor if I began to experience new period symptoms that I found difficult to manage. | 1.00 |
| I know which painkillers are most effective for period pain. | 0.83 |
| I make sure that I get information about my period from a reliable source | 1.00 |
| I double check information about the menstrual cycle to make sure that it’s reliable. | 0.83 |
| I am able to differentiate between accurate and inaccurate information about menstrual health | 1.00 |
| *I often get confused when trying to access information about my period | 1.00 |
| I know how to access accurate information about my period. | 0.83 |
| *I am uncomfortable discussing menstruation publicly | 0.83 |
| *I am uncomfortable discussing menstruation privately with other people who menstruate | 0.83 |
| I often discuss various aspects of the menstrual cycle with my friends | 1.00 |
| I know what questions to ask the doctor about my period. | 1.00 |
| If someone asked me questions about the menstrual cycle, I am confident that I could answer them accurately. | 1.00 |
| I am comfortable talking about menstruation | 0.83 |
| *I don’t know whether my period is normal | 1.00 |
| Menstrual health is an integral part of general health. | 1.00 |
| *I don’t think that period symptoms can impact academic or professional performance | 0.83 |
| *Menstrual health is not related to my overall quality of life | 0.83 |
| *I don’t think there is a need for people without periods to learn about the menstrual cycle | 1.00 |
| I understand that there are a wide variety of period symptoms e.g. abdominal cramps, memory lapse, hot flushes, pelvic pain, vaginal dryness. | 1.00 |
| Menstrual cycles change as you age | 1.00 |
| Periods usually become more regular and predictable in your 20s. | 0.83 |
| *It isn’t possible to get pregnant or already be pregnant if I'm on my period. | 1.00 |
| It is normal for your period to vary slightly (by a couple of days) from month to month | 1.00 |
| I was well prepared for my first period | 1.00 |
| I know that the average menstrual cycle is approximately 28 days long | 1.00 |
| I know that a typical period is usually between 2 and 7 days long | 1.00 |
| I understand that the menstrual cycle can vary from person to person | 1.00 |
| It is normal to feel overwhelmed around your period. | 1.00 |
| Tracking my cycle is important, it allows me to understand the changing needs of my body throughout the different stages | 1.00 |
| I make sure to change my tampon every 4-8 hours, even if it doesn’t feel like I need to | 0.83 |
| *It is okay to use a higher absorbency tampon so that I don’t have to change my tampon as often | 0.83 |
| *I wouldn’t be worried if I stopped getting periods if I could be certain that I wasn’t pregnant. | 1.00 |
| I adjust my day-to-day life (e.g. exercising, socialising, eating, sleeping) in line with when I get my period. | 0.83 |
| I know that heat, yoga and massage can be effective for managing period pain. | 0.83 |
| *It is normal to miss days of work/university because of period pain or other period symptoms | 1.00 |
| It is vital that people with periods are knowledgeable about their menstrual cycle. | 0.83 |
| *I don’t think that my diet affects my menstrual cycle | 0.83 |
| *My period doesn’t affect the way my body looks or feels | 0.83 |
| There is professional support regarding management of my period available to me if I need it. | 0.83 |
| *I would never tell my employer that I was on my period, even if it was affecting my performance or attendance in work. | 0.83 |
| *Periods are dirty | 0.83 |
| *The menstrual cycle was not properly explained to me in school | 1.00 |
| *Information about periods has to come from a healthcare professional in order to be reliable | 1.00 |
| I understand how my period is affected by external factors such as smoking, diet, exercise, stress, weight and pregnacy history. | 1.00 |
| I would consider treatments such as acupuncture, massage and reiki to reduce period pain. | 0.67 |
| I always prioritise self-care and getting adequate rest around my period | 0.67 |
| It is normal if my ability to concentrate in work/university is affected around my period. | 0.67 |
| The best way to deal with period blood differs from person to person | 0.50 |
| I feel overwhelmed by the amount of information there is about periods. | 0.67 |
| I want to learn more about the menstrual cycle | 0.67 |
| It is easy for me to access reliable information on the menstrual cycle | 0.67 |
| I have someone in my life who can answer my questions about the menstrual cycle. | 0.67 |
| I think my friends are exaggerating if they complain about period symptoms that I find easy to deal with | 0.67 |
| I believe that I know more about periods than my friends do. | 0.67 |

*Note: Reverse scored items are denoted with *, items with an I-CVI<0.78 were eliminated.*
